# Pathogenicity and whole-genome analysis of a *Siniperca chuatsi*-derived *Nocardia seriolae* strain

**DOI:** 10.3389/fmicb.2025.1623741

**Published:** 2025-08-25

**Authors:** Liting Chen, Xin Yan, Yongju Luo, Zhuanling Lu, Xinxian Wei, Zhanyang Tang, Liqun Xia, Fuyan Chen, Ming Li, Zhongbao Guo, Zhichang He, Ting Huang

**Affiliations:** ^1^Guangxi Key Laboratory of Aquatic Genetic Breeding and Healthy Aquaculture, Guangxi Academy of Fishery Science, Nanning, Guangxi, China; ^2^Key Laboratory of Comprehensive Development and Utilization of Aquatic Germplasm Resources of China (Guangxi) and ASEAN (Co-Construction by Ministry and Province), Ministry of Agriculture and Rural Affairs, Nanning, Guangxi, China; ^3^Guangdong Provincial Key Laboratory of Aquatic Animal Disease Control and Healthy Culture, Fisheries College of Guangdong Ocean University, Shenzhen Institute of Guangdong Ocean University, Guangdong, China

**Keywords:** *Siniperca chuatsi*, *Nocardia seriolae*, pathogenicity, whole-genome analysis, antibiotic susceptibility testing

## Abstract

A bacterial strain (No. 20230510) was isolated from the kidneys of diseased *Siniperca chuatsi* in Guangxi, China, since 2023. Artificial infection experiments demonstrated that this strain caused the observed disease in *S. chuatsi*. The isolate underwent morphological, pathological, genomic (whole-genome sequencing, WGS), and antibiotic susceptibility analyses. Infection trials revealed 100% mortality in high-concentration groups, with an LD50 of 3.89 × 104 CFU/mL, indicating high virulence. WGS results showed a circular chromosome of 8,123,106 bp (GC content: 68.14%), containing 7,638 CDSs, 72 tRNAs, and 12 rRNAs. Phylogenomic analysis revealed that strain 20230510 (CP130742) clusters with three *N. seriolae* strains with 98% bootstrap supporting, confirming its identification as *N. seriola*e. Further analysis identified 403 potential virulence genes linked to nutrient metabolism, regulatory factors, immune modulation, effector delivery systems, and exotoxins. Chromosomal comparisons also detected multiple antibiotic resistance genes. Susceptibility testing confirmed sensitivity to nine antibiotics, including enrofloxacin, doxycycline, florfenicol, and sulfamethoxazole. Histopathology revealed chronic granulomatous lesions, most severe in the kidneys, with similar but milder damage in the liver, spleen, gills, and intestines. These results confirm *N. seriolae* strain 20230510 as the pathogenic agent behind *S. chuatsi* mortality, offering key insights for developing control strategies.

## 1 Introduction

*Siniperca chuatsi*, a commercially important fish species in China, is prized for its tender meat and high nutritional value, driving substantial consumer demand. Official statistics report annual aquaculture production exceeding 400,000 tons (Ministry of Agriculture and Rural Affairs Fisheries Administration Bureau et al., 2023). However, intensified farming practices have led to rising disease prevalence. Documented pathogens include infectious spleen and kidney necrosis virus, iridovirus, *Aeromonas hydrophila, A. veronii, Streptococcus uberis, Flavobacterium columnare*, and *A. salmonicida* (Chen et al., 2018; Lin et al., 2020; Liu et al., 2018; Luo et al., 2017; Liu et al., 2020; Zhou et al., 2015; Zhu et al., 2023). Notably, no documented cases of *N. seriolae* infection in *S. chuatsi* have been reported to date, despite its known capability to induce mass mortality and severe economic losses in aquaculture systems.

*N. seriolae* is an environmentally ubiquitous opportunistic pathogen, with 113 *Nocardia* species identified across diverse niches, including soil, aquatic systems, rhizospheres, insects, fish, and human clinical specimens (Han et al., 2019). *Nocardiosis*, a chronic systemic disease characterized by high fatality rates (Zhang et al., 2022), primarily presents as cutaneous ulcers and visceral granulomatous lesions (Chen et al., 2000; Huang et al., 2021; Kim et al., 2018; Liao et al., 2021; Shimahara et al., 2008). First isolated from Japanese amberjack in 1968 (Kariya et al., 1968), *Nocardia* spp. have since been documented in numerous aquatic species, including *Channa maculata, Micropterus salmoides* (Chen, 1992), *Crassostrea gigas* (Friedman et al., 1998), *Oncorhynchus nerka* (Isik et al., 1999), *Terapon jarbua* (Wang et al., 2009), *Trachinotus blochii* (Vu-Khac et al., 2016), *Anguilla japonica* (Kim et al., 2018), *Sciaenops ocellatus* (Rio-Rodriguez et al., 2021), hybrid snakehead (Zhang et al., 2022), *Oreochromis niloticus, Chanos chanos*, and *Lates calcarifer* (Nazareth et al., 2024). Among these, *N. seriolae* constitutes the predominant pathogen (Kim et al., 2018; Nazareth et al., 2024; Rio-Rodriguez et al., 2021; Vu-Khac et al., 2016; Wang et al., 2009; Zhang et al., 2022), alongside *N. asteroides* (Chen, 1992), *N. crassostrea* (Friedman et al., 1998), and *N. salmonicida* (Isik et al., 1999). Aquacultural outbreaks caused by *N. seriolae* exhibit cumulative mortality rates ranging from 0.86 to 70%, posing substantial economic risks (Chen et al., 2000; Huang et al., 2021; Kim et al., 2018; Liao et al., 2021; Matsumoto et al., 2017; Rio-Rodriguez et al., 2021; Wang et al., 2009).

The advancement of whole-genome sequencing (WGS) technologies has enabled comprehensive analyses of bacterial virulence mechanisms, host-pathogen-environment interactions, and strain-specific traits (Strauss and Falkow, 1997). As a pivotal tool for virulence factor research, WGS has been applied to characterize *N. seriolae* genomes, including virulence determinants, antimicrobial resistance genes (Han et al., 2019; Kim et al., 2021; Umeda et al., 2023; Yasuike et al., 2017), and draft assemblies (Xia et al., 2015).

In May, 2023, a disease outbreak causing significant mortality in *S. chuatsi* was documented at an aquaculture facility in Guangxi, China. Laboratory investigations identified *N. seriolae* as the causative agent through (1) histopathological concordance with nocardial infections, and (2) 16S rRNA gene sequencing (>99% identity with N. seriolae type strain). To our knowledge, no previous studies have reported *N. seriolae* infections in this fish species. In this study, we performed histopathological examinations and whole-genome sequencing (WGS) analysis of the isolated bacterium, comparing it with previously reported *N. seriolae* genomes. Using multiple databases, we classified and annotated the genome sequence of this strain, predicted gene functions, and identified virulence and resistance-related genes. These findings provide foundational data for understanding *N. seriolae's* pathogenic mechanisms in *S. chuatsi* and support the development of disease prevention strategies.

## 2 Materials and methods

### 2.1 Ethical statement

The research adhered to China's regulations on the ethical treatment and utilization of laboratory animals. The experimental protocols were reviewed and authorized by the Animal Ethics Committee of Guangxi Academy of Fisheries Sciences (Approval No. GAFS2021001).

### 2.2 Experimental materials

Diseased mandarin fish (*S. chuatsi*) specimens (mean ± SD = 76 ± 10.46 g) were collected from a commercial aquaculture facility in Nanning, Guangxi Province. Healthy specimens (50 ± 8.12 g) were procured from the Guangxi Aquatic Science Research Institute's Wuming experimental base, undergoing a 7-day acclimation period before experimentation.

### 2.3 Isolation and identification of bacteria from diseased *S. chuatsi*

Following sterile protocols, *S. chuatsi* specimens were surface-sterilized using 75% ethanol and aseptically dissected to expose muscle tissue. Bacterial isolation involved streaking tissue samples onto blood agar plates (10% sheep blood agar plates, Beijing Luqiao Technology Co., Ltd.) with subsequent 25°C incubation for 3–5 days. Morphologically distinct colonies were isolated through serial purification, yielding strain 20230510. This strain was then cultured in tryptic soy broth (25°C, 120 rpm, 5–7 days). An aliquot was subjected to Gram staining per manufacturer's protocol (Gram Stain Kit, Beijing Solarbio Science & Technology Co., Ltd.) for morphological analysis, while the remainder was allocated for virulence assessment and cryopreservation (−86°C in 20% glycerol medium).

### 2.4 Pathogenicity testing of isolated strain on *S. chuatsi*

Healthy mandarin fish (*S. chuatsi*) were acclimatized in controlled aquarium systems for 1 week prior to experimental procedures. Infection challenges were implemented in 200-L tanks across a 20-day exposure period, with test subjects stratified into five cohorts (4 experimental + 1 control; *n* = 20/group). Quadruplicate bacterial suspensions (1.24 × 107 CFU/mL) prepared in sterile saline were administered via intraperitoneal (IP) injection (0.2 mL/fish), while controls received equivalent volumes of saline. Aquaria maintenance included semiweekly 50% water renewal with continuous aeration, temperature regulation (20–28°C), and scheduled feeding (2% BW/day). Daily monitoring documented clinical signs and mortality events, with moribund specimens undergoing pathogen recovery. LD50 values with 95% confidence intervals were determined by probit regression (GraphPad Prism 9.5), with supplementary verification using the classical Reed-Muench method.

### 2.5 Whole-genome sequencing and analysis

#### 2.5.1 Molecular identification of isolated strain

Genomic DNA was extracted using the DNAzol™ Reagent DNA extraction kit (Hangzhou BoRi Technology Co., Ltd.). PCR reactions and subsequent sequencing were performed according to the methods described in reference (Heuer et al., 1997). The universal primers for 16S rRNA gene amplification and PCR reagents were all supplied by Takara Biotechnology (Dalian) Co., Ltd. The sequencing results were subjected to BLAST comparison in the NCBI database.

#### 2.5.2 Sequencing

The target strain (20230510) was cultured in tryptic soy broth (TSB) at 25 °C for 7 days under standard conditions. Genomic DNA was extracted using DNAzol™ Reagent, resuspended in 100 μL of ultrapure water, and treated with RNase A (20 μg/mL) to remove residual RNA. DNA quality was evaluated through multiple approaches, including visual inspection for particulate contamination, agarose gel electrophoresis to assess integrity, NanoDrop™ spectrophotometry for purity (A260/A280 ratio), and Qubit™ fluorometry for accurate quantification.

For Oxford Nanopore sequencing, high-quality DNA was subjected to size selection using magnetic beads. The selected DNA was then end-repaired and purified, followed by barcoding using the EXP-NBD104/114 kit. A second round of purification was performed before ligating sequencing adapters with the SQK-LSK109 kit. The final library was quantified using a Qubit™ fluorometer and loaded onto a PromethION flow cell for single-molecule real-time sequencing.

For DNBSEQ-T7 sequencing, DNA was fragmented using a Covaris ultrasonicator to produce fragments between 150 and 300 bp. The library construction included terminal polishing, addition of A-overhangs, and ligation of adapters, followed by PCR amplification. After passing quality control, the amplified libraries were denatured and circularized. DNA nanoballs (DNBs) were generated through rolling circle amplification and loaded onto patterned nanoarrays for 150 bp paired-end sequencing using the DNBSEQ-T7 platform.

#### 2.5.3 Genome assembly

Oxford Nanopore sequencing datasets underwent initial quality filtering through LongQC v1.2.0c, implementing a Q-score threshold of ≥7 for read retention. Concurrently, Illumina short-read data were processed via fastp v0.23.2 with standard parameters. *De novo* genome assembly was conducted using Flye v2.8, followed by iterative polishing cycles integrating both sequencing platforms (ONT + Illumina) to achieve high-fidelity consensus sequences.

#### 2.5.4 Gene annotation and functional clustering analysis

Genome annotation was executed with Prokka v1.14.5 for structural prediction and primary functional characterization. Functional profiling included: (1) NCBI NR database queries through protein homology searches; (2) COG functional classification via eggNOG-mapper (evolutionary genealogy framework); (3) KEGG pathway mapping using orthology-based prediction. Pathogenicity assessment involved BLASTp alignment against the Virulence Factor Database (VFDB), while antimicrobial resistance determinants were identified through CARD database interrogation using DIAMOND alignment (*E*-value < 1e-5).

#### 2.5.5 Phylogenomic tree construction

The genome sequence data was uploaded to the Type (Strain) Genome Server (TYGS) for a whole genome-based phylogenomic tree construction (Meier-Kolthoff and Göker, 2019). All pairwise comparisons among a set of *Nocardia* genomes were conducted using GBDP and accurate intergenomic distances inferred under the algorithm “trimming” and distance formula *d*_5_ (Meier-Kolthoff et al., 2013). Hundred distance replicates were calculated each. The resulting intergenomic distances were used to infer a balanced minimum evolution tree with branch support via FASTME 2.1.6.1 including SPR postprocessing (Lefort et al., 2015). Branch support was inferred from 100 pseudo-bootstrap replicates each. The tree was rooted at the midpoint (Farris, 1972) and visualized with PhyD3 (Kreft et al., 2017).

### 2.6 Histopathological observations

Pathological specimens (liver, spleen, kidney, gill, intestine) from infected mandarin fish underwent 24-h fixation in 4% paraformaldehyde (PFA). Standard histological processing included graded ethanol dehydration, paraffin embedding, and microtome sectioning (5 μm thickness). H&E-stained sections were mounted with DPX resin for bright-field microscopy analysis (Nikon Eclipse Ci-L system) with digital image acquisition via NIS-Elements software (v5.02.03).

### 2.7 Antibiotic susceptibility testing

Antimicrobial susceptibility profiling was conducted via standardized Kirby-Bauer assay. Bacterial lawns were prepared by aseptic inoculation of 200 μL adjusted suspension (0.5 McFarland) onto blood agar surfaces, followed by precision placement of antibiotic discs (Hangzhou Tianhe Microbial Reagents Co., Ltd.) using sterile forceps. Plates were subjected to standardized incubation parameters (25°C, 7 days) with subsequent quantitative assessment of inhibition zones using digital caliper measurements (Mitutoyo 500-196-30), determining *N. seriolae* 2023050's resistance profile.

## 3 Results

### 3.1 Strain isolation

**Figure 1:**
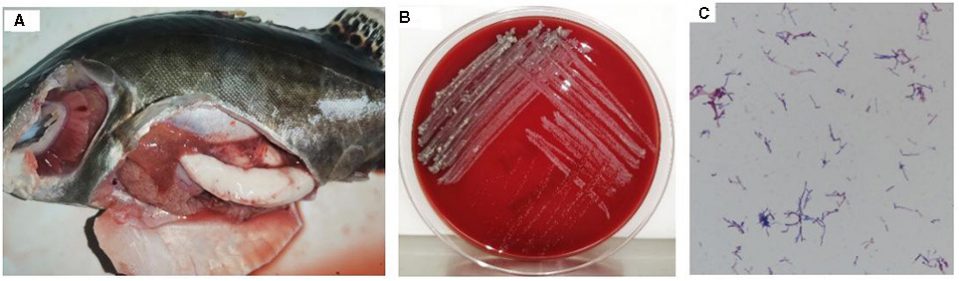
Diseased mandarin fish and bacterial morphology of strain 20230510. Diseased mandarin fish **(A)**; colony morphology on the blood plates **(B)**; Gram staining of strain 20230510 [**(C)**, 1000×].

Isolate 20230510 demonstrated extended lag-phase growth (3–5 days) on blood agar, forming pale yellow, granular colonies with irregular margins and rugose surface topography (Figure 1B). Microscopic analysis revealed Gram-positive staining with characteristic filamentous branching morphology (Figure 1C).

### 3.2 Pathogenicity testing

Healthy *S. chuatsi* were challenged with *N. seriolae* strain 20230510. High-dose groups (1.24 × 106−1.24 × 107 CFU/fish) exhibited behavioral abnormalities (lethargy, anorexia, delayed responsiveness) by day 3 post-infection, with mortality initiating on day 8 (Table 1). Comparatively, groups receiving 1.24 × 104−1.24 × 106 CFU showed delayed mortality (days 10–13). By day 16, all high-dose fish succumbed, while only four mortalities occurred in the low-dose group (1.24 × 104 CFU/fish) by day 20. No mortality was observed in controls over 21 days. The calculated LD50 was 3.89 × 104 CFU/mL.

**Table 1:** Results of artificial infection of *Siniperca chuatsi*.

**Concentration for challenging/CFU/mL**	**Experim-ental fish no**.	**Mortality no**.										**Accumulated no. of deaths**	**Mortality (%)**
		**8d**	**9d**	**10d**	**11d**	**12d**	**13d**	**14d**	**15d**	**16d**	**17d**		
1.24 × l0^7^	20	1	2	1	3	7	4	2	0	0	0	20	100%
1.24 × l0^6^	20	0	0	1	1	3	5	6	3	1	0	20	100%
1.24 × l0^5^	20	0	0	0	1	2	1	3	6	2	1	16	80%
1.24 × l0^4^	20	0	0	0	0	0	1	1	2	0	0	4	20%
Control group	20	0	0	0	0	0	0	0	0	0	0	0	0

Affected fish lacked skin ulcers but displayed fin congestion/ulceration (dorsal, pelvic, anal). Necropsy identified abdominal distension, ascites, and posterior kidney enlargement (Figure 1A), mirroring natural infection phenotypes. Control specimens remained asymptomatic. Reisolated bacteria from experimental mortalities matched natural infection isolates in purity, colony morphology, and growth kinetics.

### 3.3 WGS results of strain 20230510

**Figure 2:**
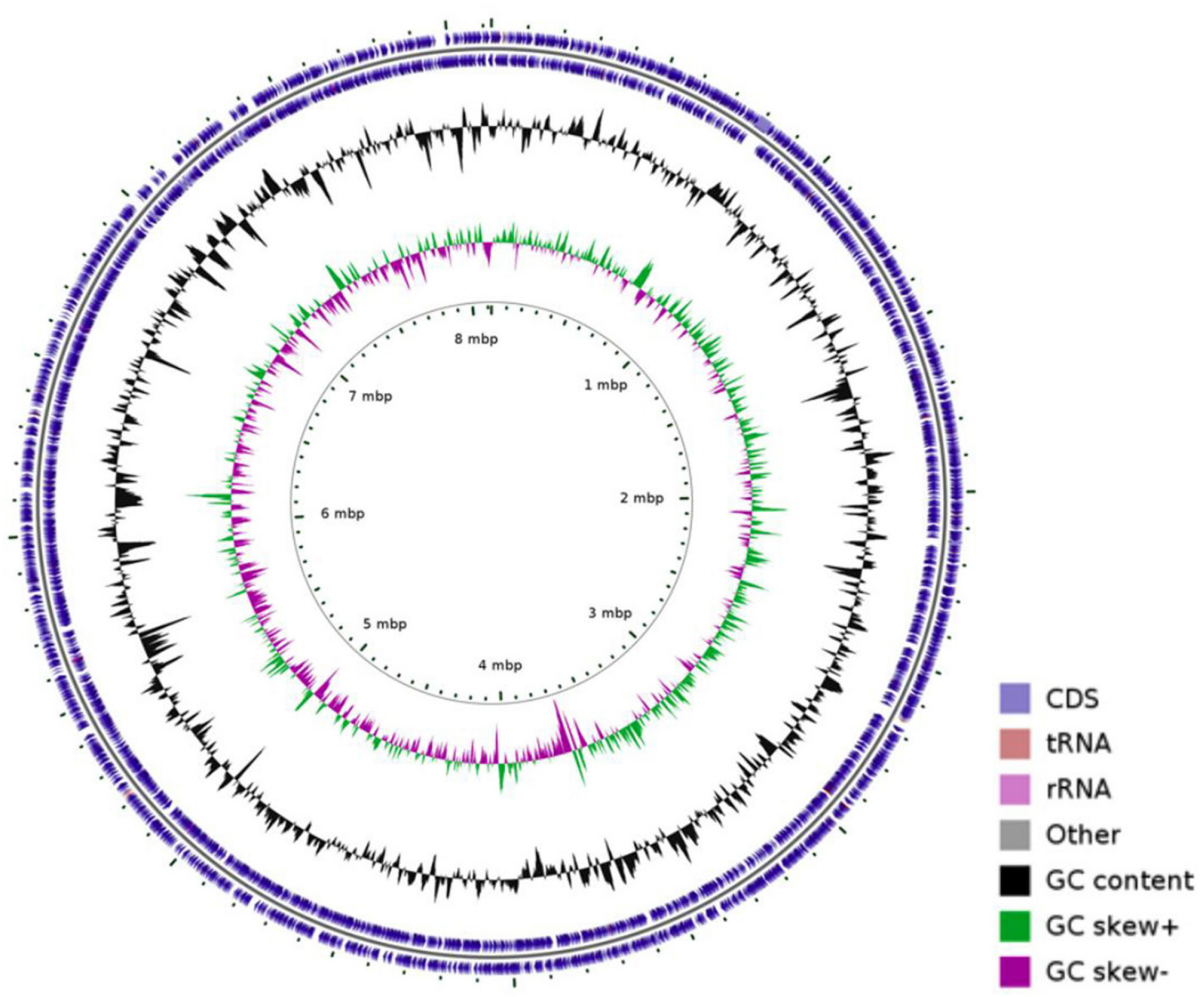
The circular genome map of strain 20230510.

Whole-genome sequencing of *N. seriolae* strain 20230510 revealed a circular chromosome (8,123,106 bp; GC 68.14%) encoding 7,638 CDSs, 12 rRNAs, 72 tRNAs, and 1 tmRNA (Figure 2). The genome (GenBank: CP130742) was compared with seven reference strains, showing conserved GC content (~68.1%) despite size variations (7.70–8.37 Mb; Table 2).

**Table 2:** Genomic features of the *N. seriolae* strain 20230510 genome and comparison with genomes of other *N. seriolae* strains.

**Strains**	**GenBank accession no**.	**Year of isolation**	**Isolation area**	**Host**	**Genome size (bp)**	**GC content (mol%)**	**No. of CDSs**	**No. of rRNA**	**No. of tRNA**	**References**
20230510	CP130742	2023	Nanning, China	*Siniperca chuatsi*	8123106	68.14%	7,638	12	72	This study
CK-14008	NZ_MOYO00000000.1 ^a^	2014	Busan, Korea	*Channa argus*	8370754	68.1%.	7,903	12	66	Han et al., 2019
EM150506	CP017839.1	2015	Gimcheon Gyeongsangbuk-do, Korea	*Anguilla japonica*	8304518	68.1%	7,794	12	65	Han et al., 2019
KGN1266	AP028458	2012	Kagoshima, Japan	*Seriola dumerili*	8222513	68.1%	8,045	12	74	Umeda et al., 2023
024013	AP028459	2002	Oita, Japan	*Seriola quinqueradiata*	8113213	68.1%	7,815	12	72	Umeda et al., 2023
UTF1	AP017900	2008	Miyazaki Prefecture, Japan	*Seriola quinqueradiata*	8121733	68.1%	7,697	4	62	Yasuike et al., 2017
ZJ0503	JNCT01000000	2005	Zhangjiang, China	*Trachinotus ovatus*	7708091	68.25%	7,426	1	62	Xia et al., 2015
MH196537	–	–	–	*Anguilla japonica*	8262437	68.1%	8,072	12	66	Kim et al., 2021

a NZ_MOYO00000000.1 is a draft genome accession number that includes a complete chromosome and two incomplete plasmids.

**Figure 3:**
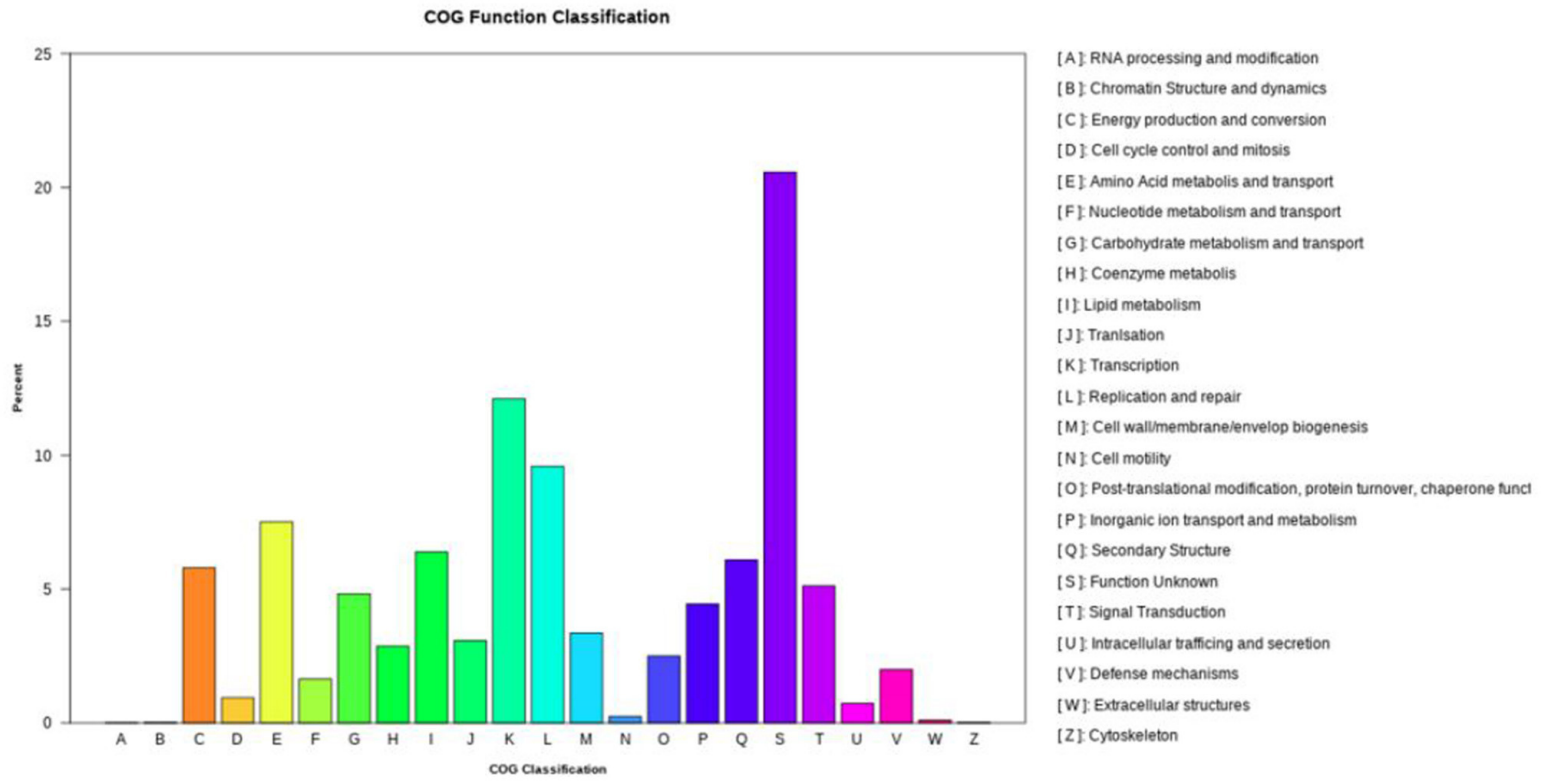
The clusters of orthologous genes (COG) functional annotation in the whole genome of *N. seriolae* strain 20230510.

COG functional annotation classified 7,108 CDSs into 23 categories (Figure 3), with dominant functional groups including transcription (Class K, 861), replication/repair (L, 681), amino acid transport (E, 534), lipid metabolism (I, 454), secondary structures (Q, 433), and energy production (C, 412). Additionally, 1,462 genes lacked functional assignments.

**Figure 4:**
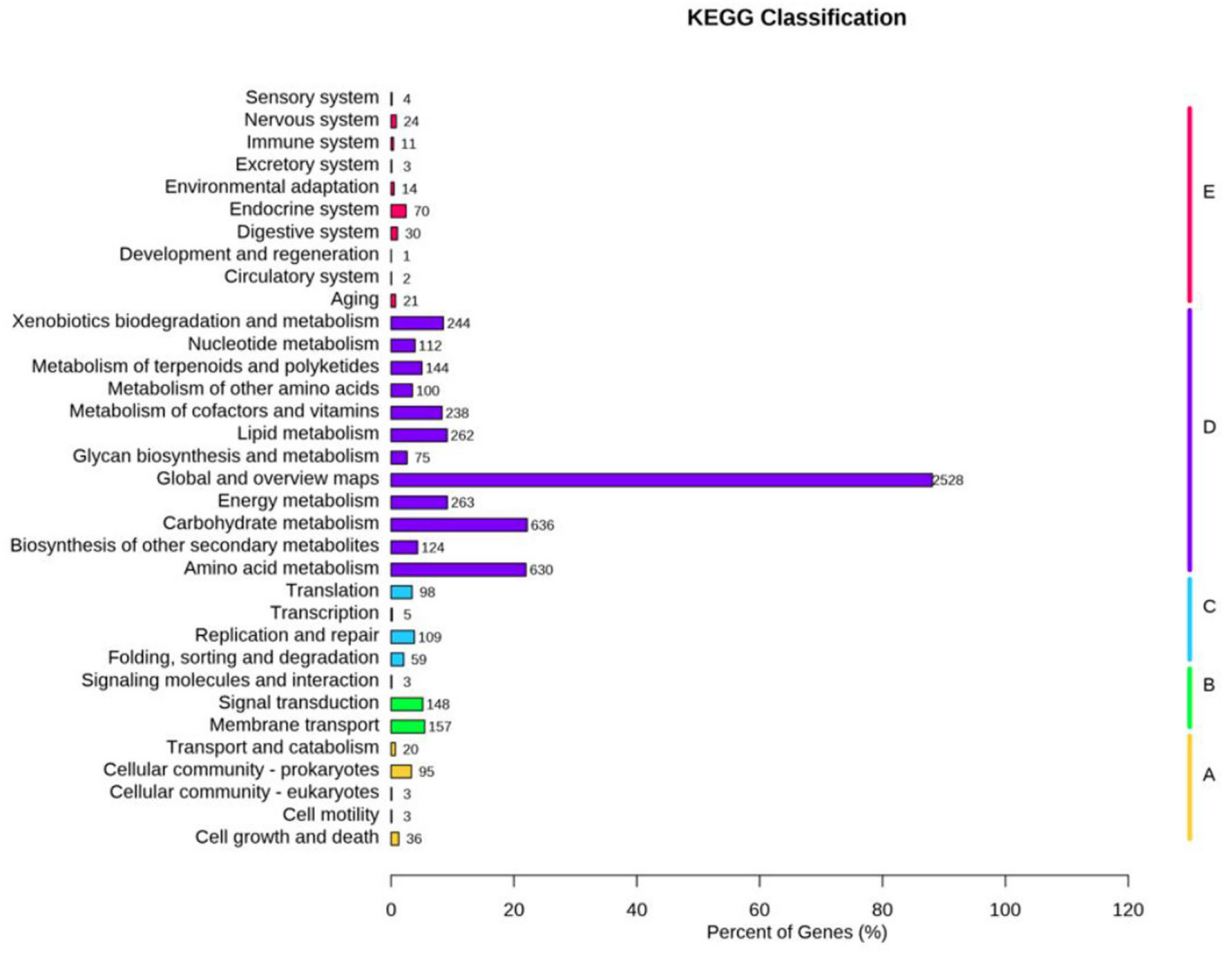
The Kyoto encyclopedia of genes and genomes (KEGG) functional annotation of *N. seriolae* strain 20230510.

KEGG pathway analysis mapped 6,272 genes to 252 metabolic processes (Figure 4), predominantly involved in core metabolism (68.3%), environmental signal transduction (15.1%), and genetic information processing (11.6%).

### 3.4 Phylogenomic tree analysis

**Figure 5:**
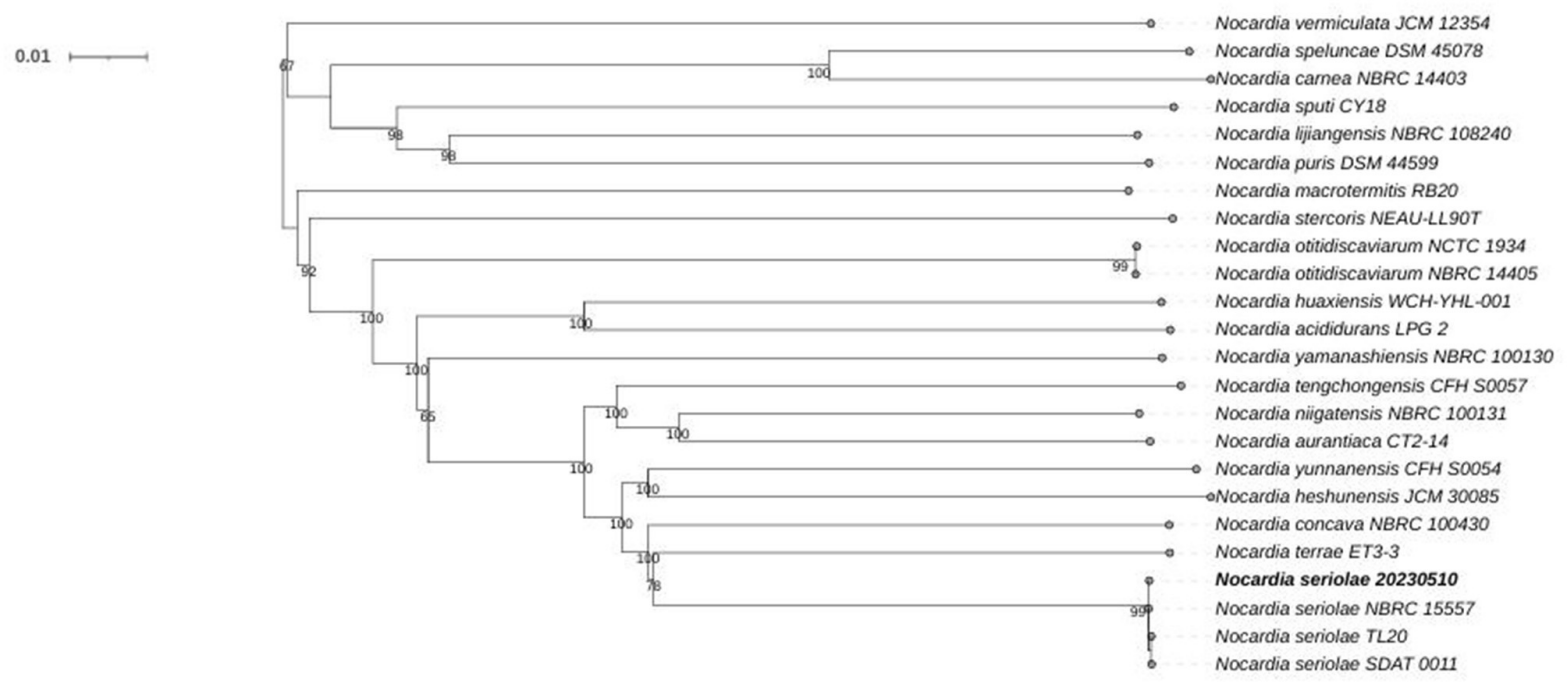
Genome—wide phylogenetic tree of 21 *Nocardia* strains.

**Figure 6:**
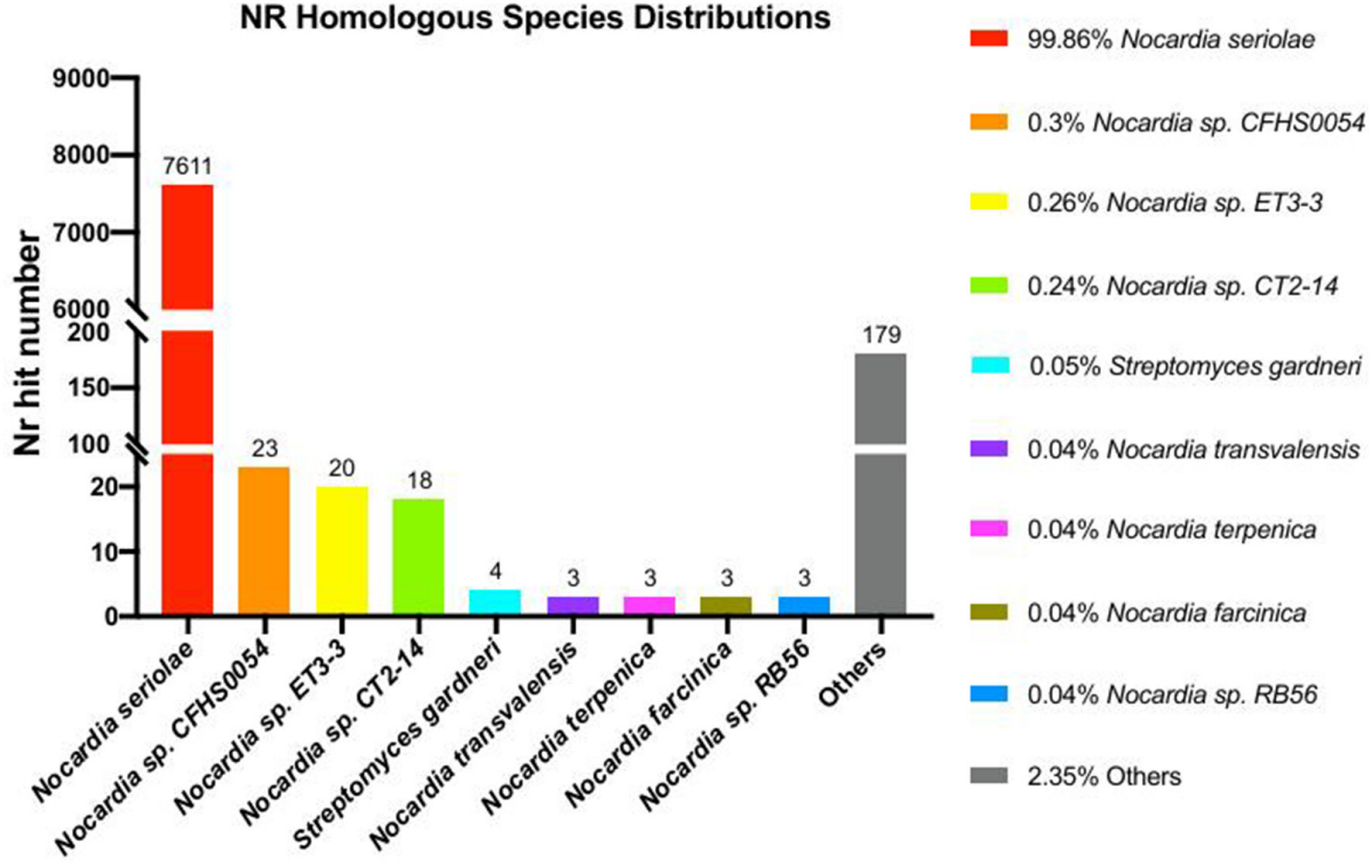
Non-redundant protein database (NR) functional annotation *N. seriolae* of strain 20230510.

Phylogenomic analysis of 24 *Nocardia* species revealed that strain 20230510 clusters within the *N. seriolae* clade, with 99% bootstrap supporting (Figure 5). NR database annotations further confirmed taxonomic alignment, with 99.96% genus-level (*Nocardia*) and 99.86% species-level (*N. seriolae*) sequence homology (Figure 6), conclusively identifying the isolate as *N. seriolae*.

### 3.5 Virulence factor analysis

**Figure 7:**
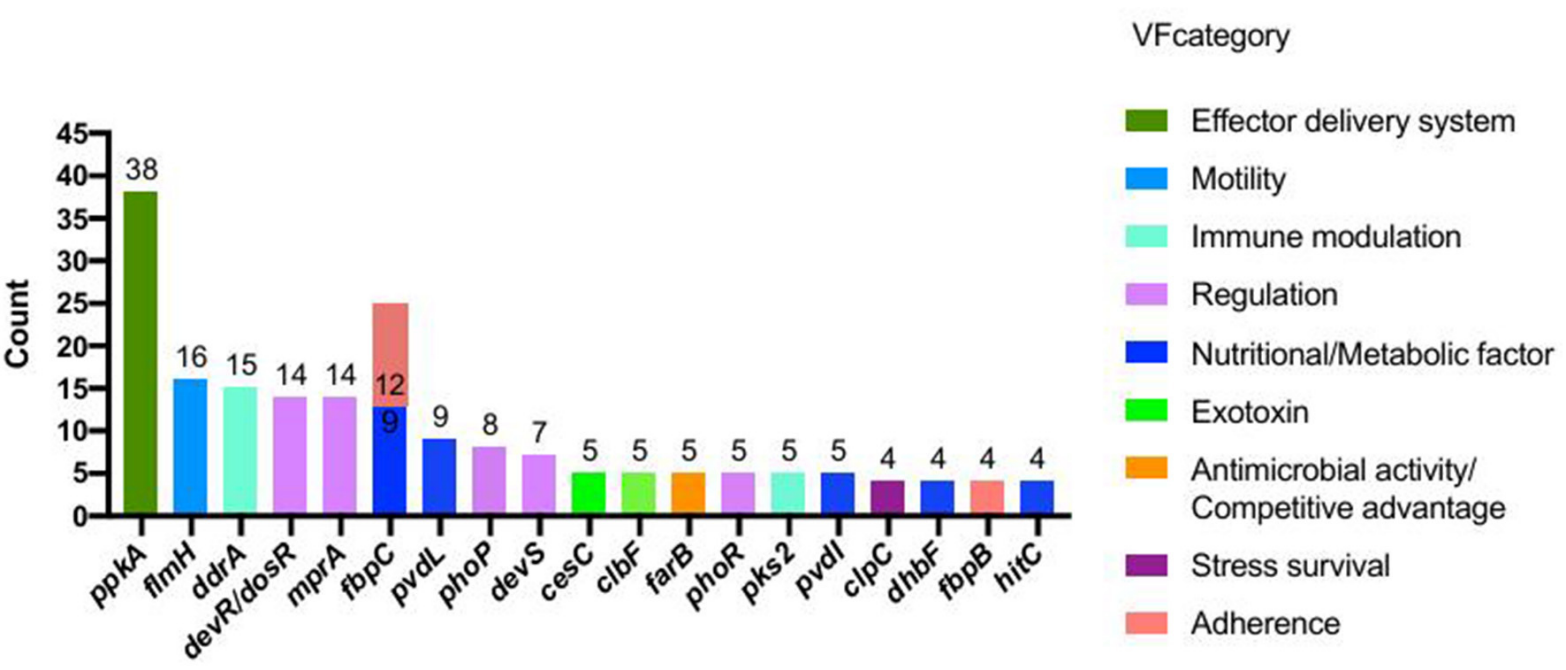
Virulence factors of *N. seriolae* of strain 20230510.

VFDB analysis identified 403 putative virulence-associated CDSs in strain 20230510 (Supplementary Table 1). These genes were functionally annotated into 12 categories, with the most prevalent involving nutrient metabolism (115 genes, e.g., fbpC, pvdL, dhbF, hitC), regulatory systems (76 genes; devR/dosR, mprA, phoP), immune modulation (63 genes; ddrA, pks2, cpsA/uppS), effector delivery (54 genes; ppkA, cdsN, eccA1-C3), and exotoxin production (28 genes; cesC, clbF/D, cyaB). The top 20 virulence factors by gene count are illustrated in Figure 7.

### 3.6 Antibiotic resistance gene analysis

**Figure 8:**
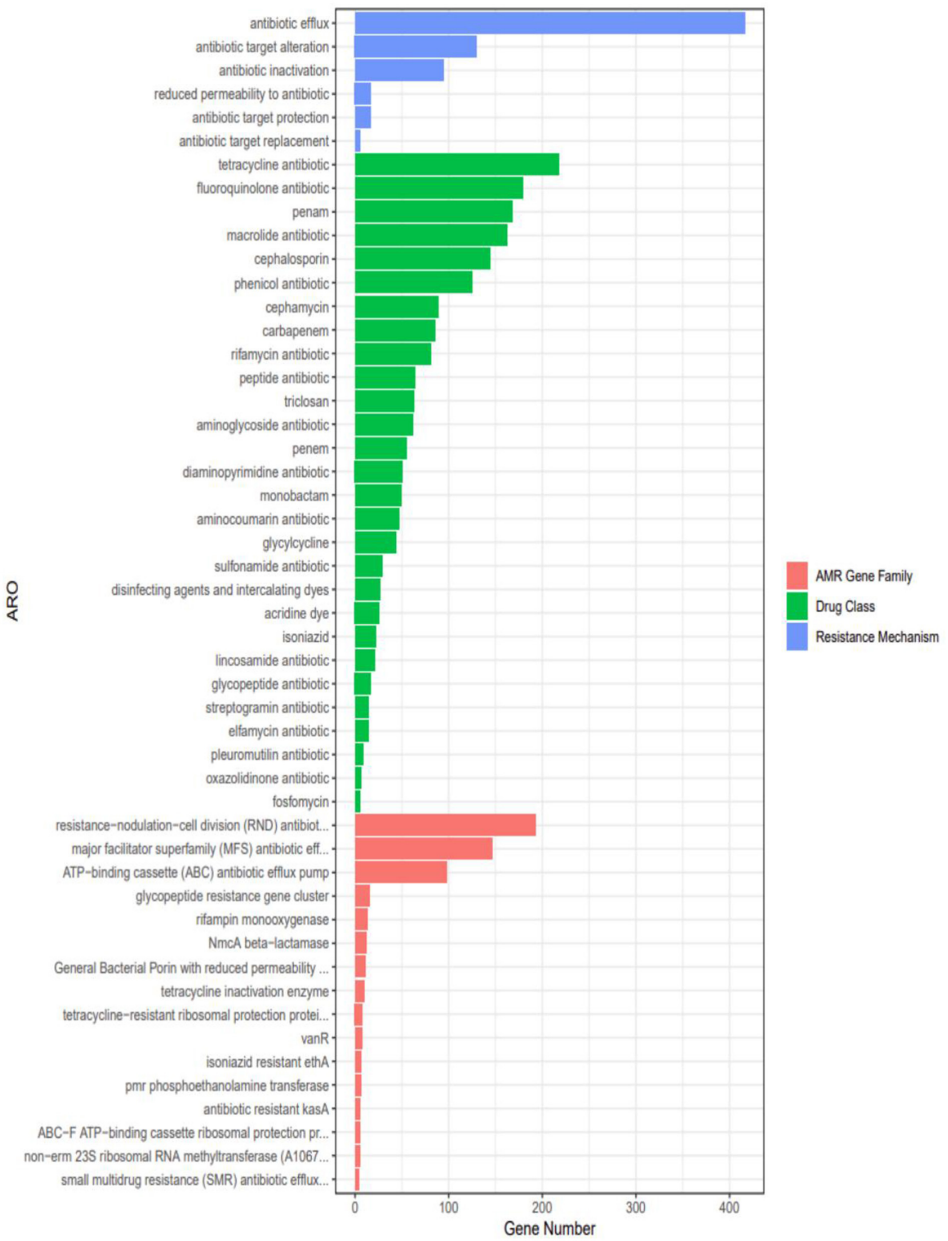
Results of drug resistance gene analysis of *N. seriolae* of strain 20230510.

Genomic analysis identified 28 classes of antimicrobial resistance (AMR) genes in strain 20230510, spanning tetracyclines, fluoroquinolones, β-lactams (penams, cephalosporins, cephamycins, carbapenems), macrolides, chloramphenicol, and rifamycins (Figure 8). Quantitative profiling revealed predominant resistance to tetracyclines (218 genes), followed by fluoroquinolone-(179 genes) and penam-targeting mechanisms (168 genes).

### 3.7 Histopathological observations

**Figure 9:**
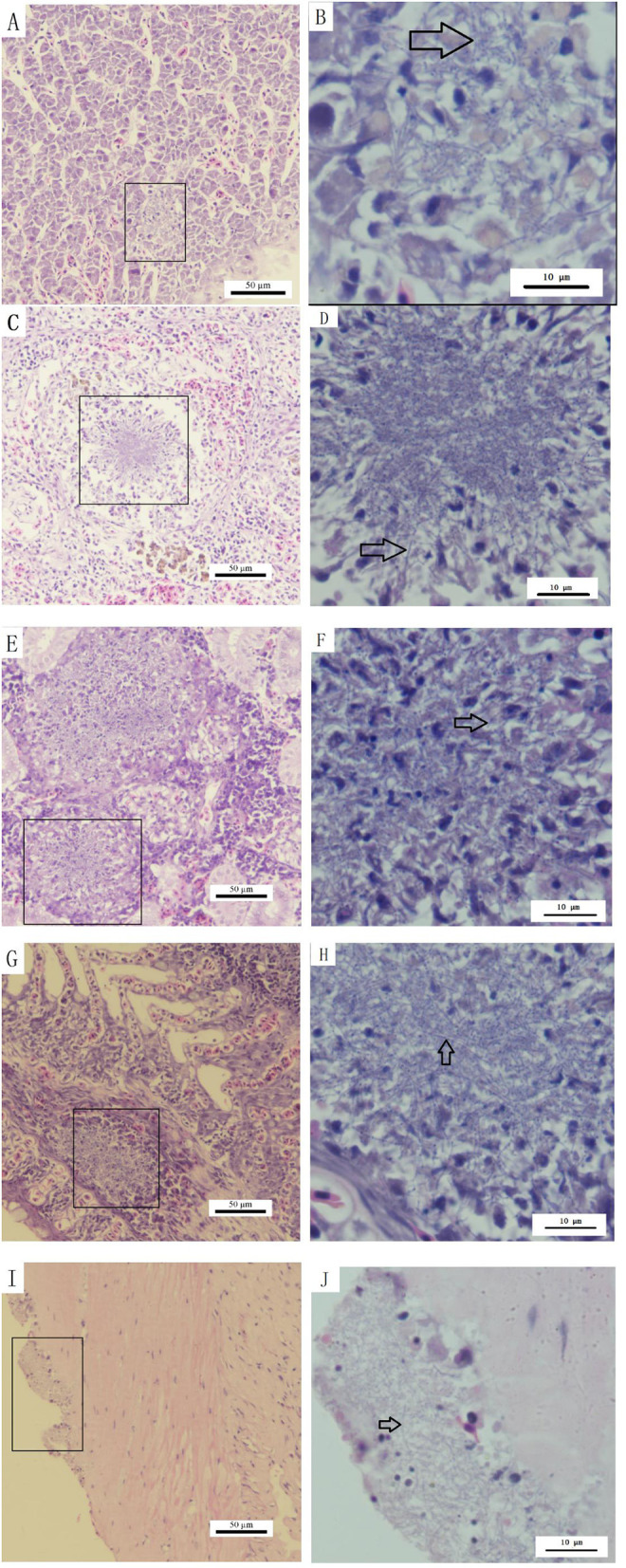
Histopathological observations of diseased *S. chuatsi*. **(A, C, E, G, I)** Show granulomas or lesion areas in or on the surface of the liver, spleen, kidneys, gills, and intestines, respectively; **(B, D, F, H, J)** provide magnified views of selected granulomas or lesion areas of **(A, C, E, G, I)**, respectively. Within these images, *N. seriolae* are indicated by arrows, and the lesion or granulomatous areas are marked by squares.

Histopathological assessment of infected *S. chuatsi* demonstrated systemic granulomatous inflammation, with acid-fast stained *N. seriolae* (blue) present in all examined organs (Figure 9). Organ-specific manifestations were as follows:

**Liver:** Hepatocellular degeneration/necrosis accompanied by developing granulomas (Figure 9A), with intralesional bacterial colonization (Figure 9B).

**Spleen:** Connective tissue hyperplasia and lymphocyte depletion (Figure 9C), featuring necrotic foci with epithelioid cell aggregates, neutrophil infiltration, and marked macrophage proliferation (Figure 9D).

**Kidneys:** Most severe granulomatous involvement, showing large-diameter lesions (Figure 9E) with interstitial necrosis and dense macrophage/neutrophil infiltration (Figure 9F).

**Gills:** Granulomatous infiltration by macrophages/lymphocytes (Figures 9G, H).

**Intestines:** Structurally intact but with surface-adherent pathogens (Figures 9I, J).

### 3.8 Antibiotic susceptibility testing

Antibiotic susceptibility profiling of strain 2023510 against 16 agents (Table 3) identified sensitivity to nine antimicrobials spanning five classes: quinolones (ciprofloxacin, norfloxacin), tetracyclines (doxycycline), macrolides (erythromycin), cephalosporins (cefoperazone), sulfonamides (florfenicol, sulfamethoxazole), and aminoglycosides (kanamycin, gentamicin). The strain demonstrated resistance to cefuroxime, polymyxin B, amoxicillin, and carbenicillin.

**Table 3:** Results of antibiotic susceptibility testing for strain 20230510.

**Antibiotic category**	**Drug**	**Drug content (μg/disc)**	**Sensitivity**
Fluoroquinolones	Enrofloxacin	10	I
Quinolones	Ciprofloxacin	5	S
Third-generation quinolones	Ofloxacin	5	I
Quinolones	Norfloxacin	10	S
Tetracyclines	Doxycycline	30	S
Macrolides	Erythromycin	30	S
Cephalosporins	Cefoperazone	75	S
Cephalosporins	Cefuroxime	30	R
Polypeptides	Polymyxin B	300	R
Broad-spectrum amide antibiotics	Florfenicol	30	S
Aminoglycosides	Neomycin	30	I
Aminoglycosides	Kanamycin	30	S
Aminoglycosides	Gentamicin	10	S
Sulfonamides	Sulfamethoxazole	300	S
Beta-lactams, Penicillins	Amoxicillin	25	R
Penicillins	Carbenicillin	100	R

Notably, four sensitive agents—enrofloxacin, doxycycline, florfenicol, and sulfamethoxazole—are approved by China's Ministry of Agriculture and Rural Affairs under the Aquaculture Medication Guidelines (2024 Documents 2). These compounds, along with ciprofloxacin, erythromycin, cefoperazone, kanamycin, and gentamicin, constitute viable therapeutic options for managing *N. seriolae* infections in *S. chuatsi*.

## 4 Discussion

Experimental infection of *S. chuatsi* with *N. seriolae* isolated from naturally diseased fish reproduced clinical signs consistent with natural infections, including fin congestion, ulceration, and ascites, though ulcerative skin lesions were absent. Histopathological analysis revealed systemic granulomatous inflammation in the liver, spleen, kidneys, intestines, and gills, with visible clusters of *N. seriolae* in affected tissues. These findings align with previous reports in other species but exhibit notable variations. For instance, Huang et al. (2021) described granulomatous necrosis and lymphoid depletion in largemouth bass, while Kim et al. (2018) documented nodular lesions in eels with myocardial necrosis and hepatic congestion. Conversely, tilapia and milkfish failed to develop granulomas (Nazareth et al., 2024), and East Asian four-finger threadfins exhibited acute necrotic lesions with purulent fluid (Liao et al., 2021). The pathological changes observed in this study do not completely align with the typical characteristics of *N. seriolae* infections in other fish species. This discrepancy may be attributed to different responses of various fish species to *N. seriolae* or the varying pathogenicity of different strains. Further comparative studies are needed to elucidate the mechanisms underlying these interspecies differences.

Traditional bacterial identification relies on a polyphasic approach combining morphological, physiological, and molecular techniques (16S rRNA and multilocus sequence analysis) to ensure taxonomic accuracy (Friedman et al., 1998; Isik et al., 1999; Rio-Rodriguez et al., 2021; Vu-Khac et al., 2016; Wang et al., 2009). However, advancements in next-generation sequencing have revolutionized microbial identification by enabling rapid whole-genome characterization. In the present study, strain 20230510 was isolated from diseased *S. chuatsi* in Guangxi aquaculture facilities. Comprehensive genomic analysis confirmed its identity as *N. seriolae* through: (1) 16S rRNA sequencing (>99% homology with *N. seriolae*), and (2) whole-genome sequencing (99.86% average nucleotide identity with *N. seriolae* reference strains). The complete genome assembly revealed a typical Nocardia genomic architecture—a single circular chromosome spanning 8.12 Mb with 68.14% GC content and 7,638 coding sequences. These genomic features align well with previous reports documenting N. seriolae genomes ranging 7.70–8.37 Mb with ~68.1% GC content and 7,426–8,072 CDSs (Han et al., 2019; Kim et al., 2021; Strauss and Falkow, 1997; Umeda et al., 2023; Yasuike et al., 2017). The minor variations observed in genome size and gene content likely reflect natural strain-to-strain genomic diversity within the species.

Vaccination is considered an effective method to control bacterial infections, and scholars have been working toward developing vaccines against *N. seriolae* (Chang et al., 2016; Chen et al., 2019; Matsumoto et al., 2017; Nayak et al., 2014; Shimahara et al., 2010; Wang et al., 2022). However, no vaccine for *N. seriolae* has yet been approved for use, and antibiotics remain the primary treatment for *N. seriolae* infections in aquaculture production. In Japan, only sulfamonomethoxine and sulfisozole sodium are licensed for the treatment of *N. seriolae* infections (Imajoh et al., 2016; Ismail et al., 2012). Liao et al. (2021) found that *N. seriolae* strains NM107152 and NM108007 were susceptible to oxytetracycline, doxycycline, lincomycin, erythromycin, and florfenicol. Chang et al. (2016) discovered that oxytetracycline, trimethoprim, erythromycin, florfenicol, and thiamphenicol were effective drugs for treating *N. seriolae* infections in fish. Imajoh et al. (2016) identified that the antibiotic resistance genes of the *N. seriolae* U-1 strain included one gene for vancomycin resistance, two for fluoroquinolone resistance, and 10 for β-lactamase resistance, indicating that the draft genome of U-1 lacks genes responsible for resistance to macrolides and tetracyclines. Yasuike et al. (2017) found that the *N. seriolae* UTF1 strain was resistant to oxytetracycline (a tetracycline antibiotic) but susceptible to erythromycin (a macrolide antibiotic). Our experimental results do not fully align with the above studies. Moreover, the antibiotic susceptibility testing results of this strain were not fully consistent with the resistance genes. These discrepancies may be due to variations in strains isolated from different regions, leading to different results in antibiotic susceptibility testing or resistance gene identification. Additionally, the discrepancies could be due to the different principles of the two testing methods: resistance genes are detected in the genome sequence as mutations of one or several drug resistance-related genes, while antibiotic susceptibility testing is a phenotypic test that measures the *in vitro* sensitivity of the bacterium to a specific drug. Furthermore, antibiotic resistance genes do not necessarily translate into an antibiotic-resistant phenotype (Zhu et al., 2023). As suggested above, the mechanisms underlying the inconsistency between resistance genes and antibiotic susceptibility results for *N. seriolae* require further in-depth investigation.

Antimicrobial susceptibility testing demonstrated that strain 20230510 exhibited sensitivity to multiple antibiotic classes, including quinolones, tetracyclines, macrolides, cephalosporins, sulfonamides, and aminoglycosides. These findings provide valuable insights for clinical management of *N. seriolae* infections in aquaculture. In accordance with the antimicrobial susceptibility profile and considering the approved veterinary drugs listed by the Ministry of Agriculture and Rural Affairs of China, we recommend the following treatment protocol for *N. seriolae* infections in *S. chuatsi*: primary options is enrofloxacin (quinolone) and doxycycline (tetracycline), and alternative choices is florfenicol (phenicol) and sulfamethoxazole (sulfonamide). This therapeutic strategy is proposed based on: demonstrated *in vitro* efficacy against the isolated strain; compliance with national regulations on veterinary drug use; practical considerations for aquaculture applications. The broad susceptibility pattern observed suggests these antimicrobials remain effective for controlling *N. seriolae* outbreaks in mandarin fish populations, though continuous monitoring of resistance development is advised.

The results of the present study indicate that *N. seriolae* is pathogenic to Mandarin fish; however, further research on the source, infection pathways, pathogenicity, and immune mechanism of the pathogen is required.

## Data Availability

The original contributions presented in the study are publicly available. This data can be found in here: https://www.ncbi.nlm.nih.gov/genbank/, CP130742.
